# Supplementary material for: Productive Performance and Egg and Meat Quality of Two Indigenous Poultry Breeds in Vietnam, Ho and Dong Tao, Fed on Commercial Feed
Source: Animals (Basel). 2020 Mar 1;10(3):408. doi: 10.3390/ani10030408 (PMC7143232; doi:10.3390/ani10030408)
Supplement: Supplementary file 1 [file animals-10-00408-s001.pdf]

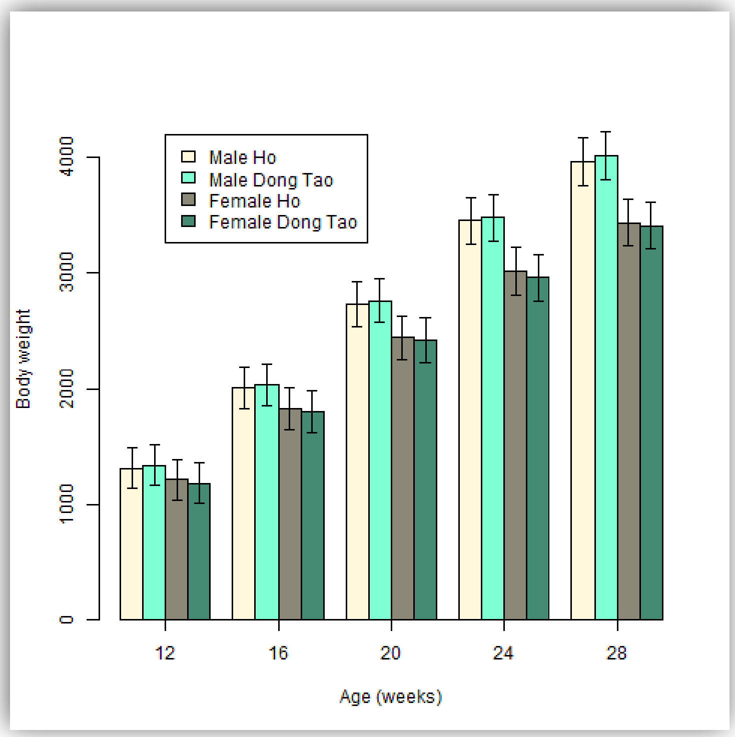

Figure S1 : Body weight as a function of breed, age and sex.

Error bars represent one standard error.

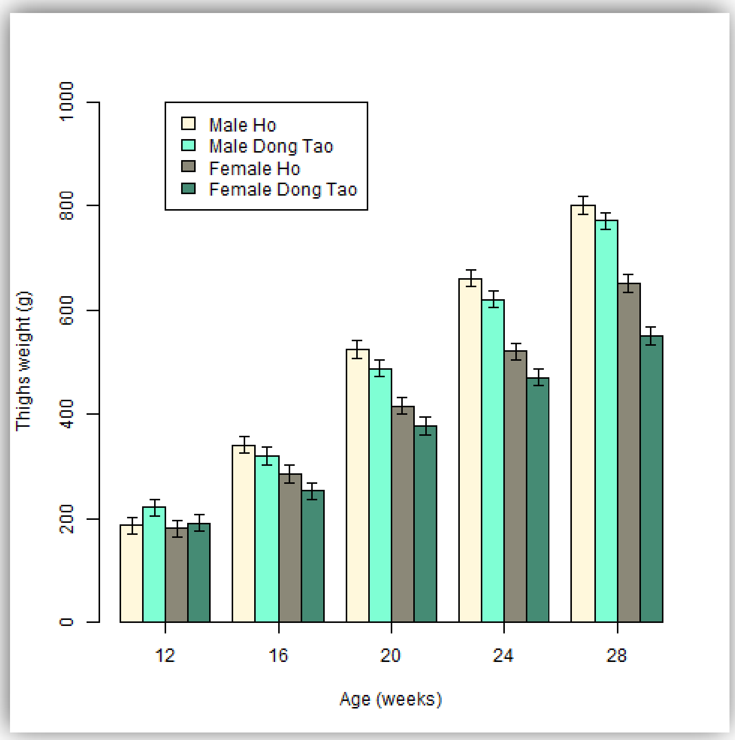

Figure S2 : Thighs weight as a function of breed, age and sex.

Error bars represent one standard error.

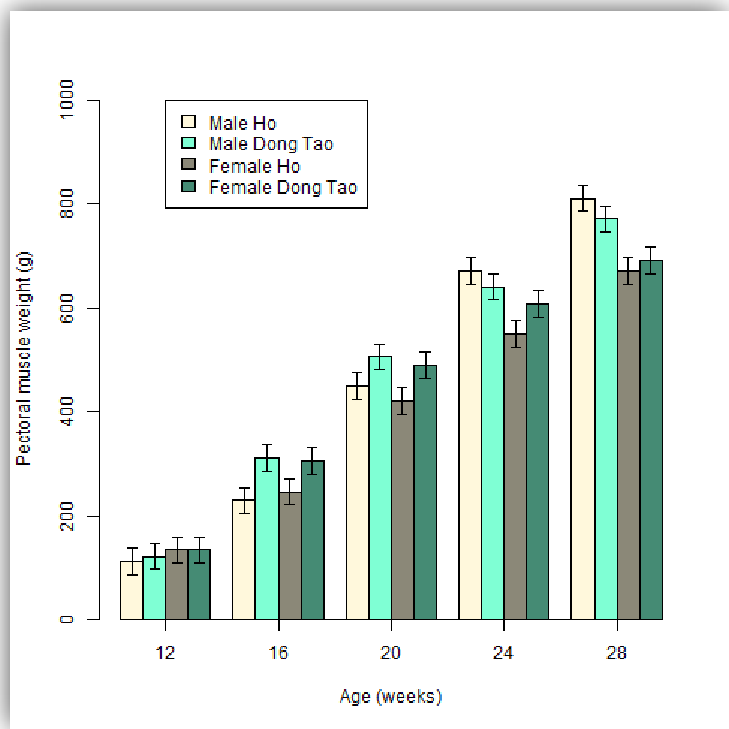

Figure S3 : Pectoral muscle weight as a function of breed, age and sex.

Error bars represent one standard error.

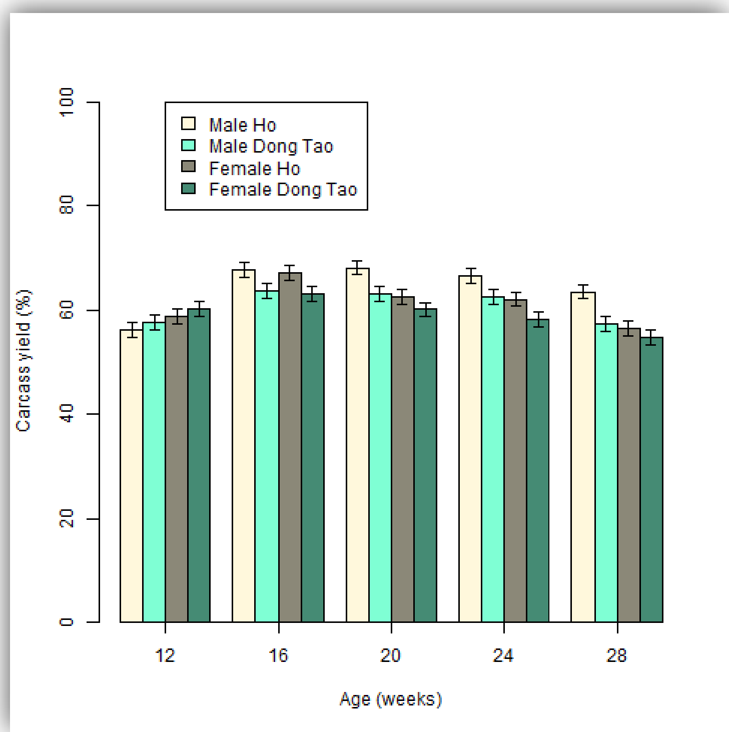

Figure S4 : Carcass yield as a function of breed, age and sex.

Error bars represent one standard error.

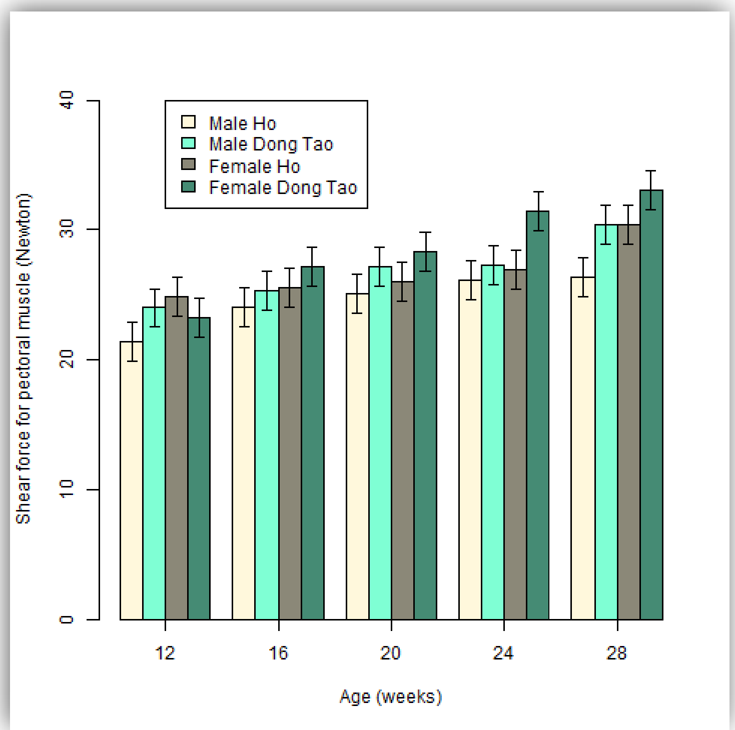

Figure S5 : Shear force for pectoral muscle as a function of breed, age and sex.

Error bars represent one standard error.

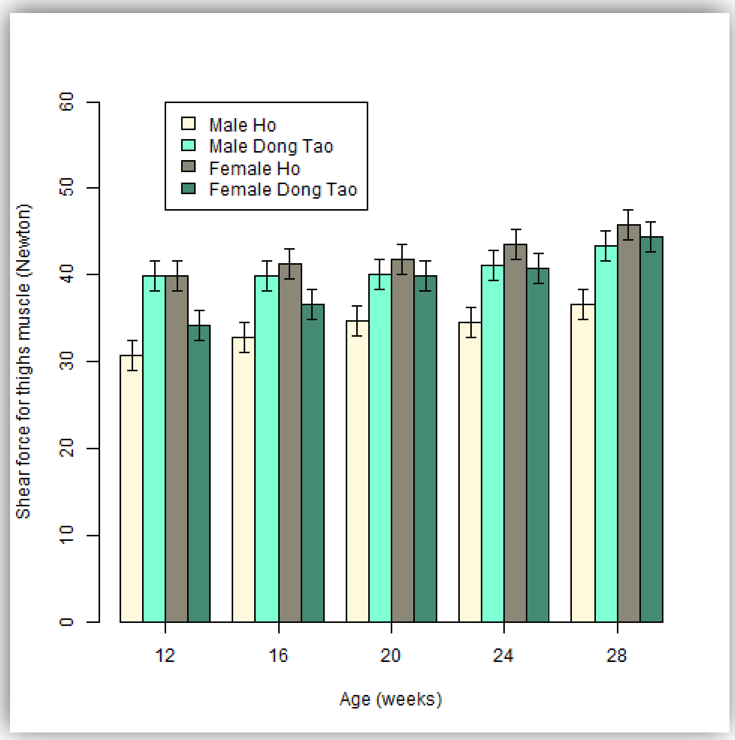

Figure S6 : Shear force for thighs muscle as a function of breed, age and sex.

Error bars represent one standard error.

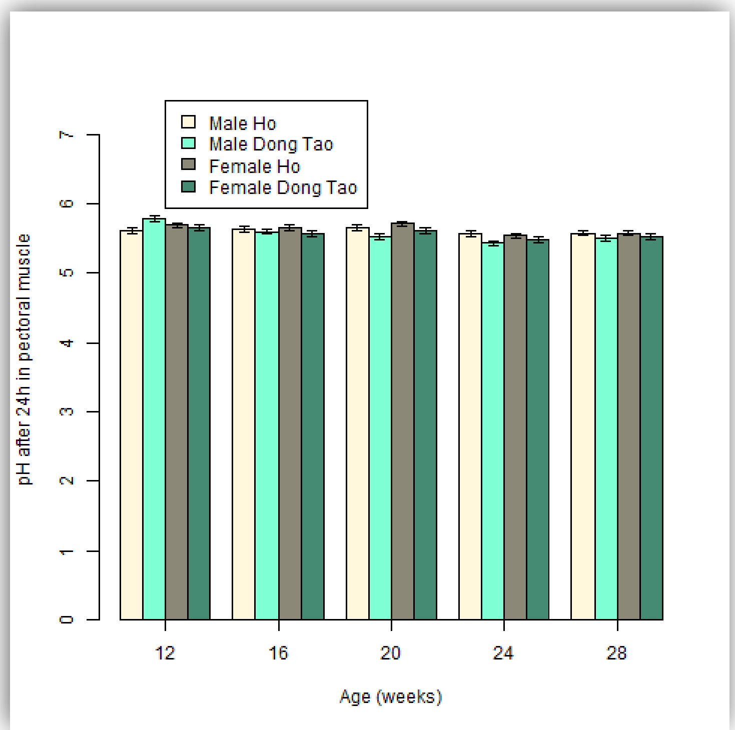

Figure S7 : pH in pectoral muscle after 24h as a function of breed, age and sex.

Error bars represent one standard error.

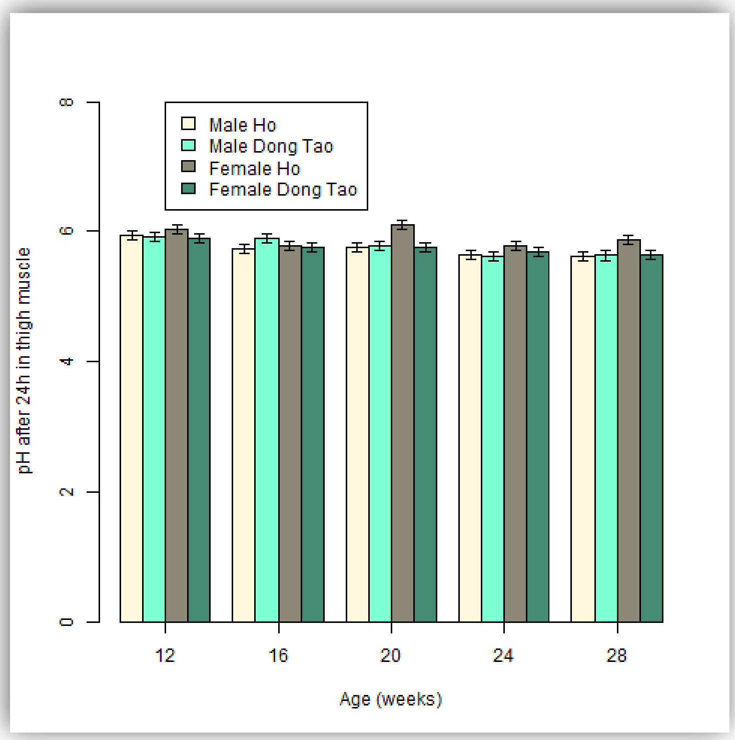

Figure S8 : pH in thighs muscle after 24h as a function of breed, age and sex.

Error bars represent one standard error.
